# Molecular Dysfunction and Phenotypic Derangement in Diabetic Cardiomyopathy

**DOI:** 10.3390/ijms20133264

**Published:** 2019-07-02

**Authors:** Isabella Evangelista, Ranuccio Nuti, Tommaso Picchioni, Francesco Dotta, Alberto Palazzuoli

**Affiliations:** 1 Cardiovascular Diseases Unit, Department of Internal Medicine, Le Scotte Hospital University of Siena, Viale Bracci, 53100 Siena, Italy; 2 Diabetes Unit, Department of Medicine, Surgery and Neurosciences, Le Scotte Hospital University of Siena, Viale Bracci, 53100 Siena, Italy

**Keywords:** heart failure, diabetes, diabetic cardiomyopathy, myocardial remodeling, molecular signal derangement

## Abstract

The high incidence and poor prognosis of heart failure (HF) patients affected with diabetes (DM) is in part related to a specific cardiac remodeling currently recognized as diabetic cardiomyopathy (DCM). This cardiac frame occurs regardless of the presence of coronary artery diseases (CAD) and it can account for 15–20% of the total diabetic population. The pathogenesis of DCM remains controversial, and several molecular and cellular alterations including myocardial hypertrophy, interstitial fibrosis, oxidative stress and vascular inflammation, have been postulated. The main cardio-vascular alterations associated with hyperglycemia comprise endothelial dysfunction, adverse effects of circulating free fatty acids (FFA) and increased systemic inflammation. High glucose concentrations lead to a loss of mitochondrial networks, increased reactive oxygen species (ROS), endothelial nitric oxide synthase (eNOS) activation and a reduction in cGMP production related to protein kinase G (PKG) activity. Current mechanisms enhance the collagen deposition with subsequent increased myocardial stiffness. Several concerns regarding the exact role of DCM in HF development such as having an appearance as either dilated or as a concentric phenotype and whether diabetes could be considered a causal factor or a comorbidity in HF, remain to be clarified. In this review, we sought to explain the different DCM subtypes and the underlying pathophysiological mechanisms. Therefore, the traditional and new molecular and signal alterations and their relationship with macroscopic structural abnormalities are described.

## 1. Introduction

Diabetes mellitus (DM) is universally considered a traditional cardiovascular (CV) risk factor but its association with heart failure (HF) is undefined. Frequently the two diseases coexist, and DM can influence the clinical evaluation, management and prognosis of patients suffering from HF [1]. The relationship linking diabetes and HF is bidirectional: diabetes increases the risk of HF and HF has a worse outcome in the presence of diabetes. DM can potentially accelerate the atherosclerotic process, endothelial dysfunction, myocardial fibrosis and oxidative stress, leading to increased coronary artery disease (CAD) burden, left ventricular (LV) hypertrophy and intrinsic myocardial cells damage [2]. A recent population-based cohort registry demonstrated that diabetes is the primary driver for combined end point of myocardial infarction (MI) and HF [3,4]. Therefore, a result from the ESC Heart Failure Registry revealed that hospitalized patients with diabetes have 1.5-fold and 3-fold increased risk for in-hospital and long-term mortality respectively, when compared with non-diabetics [5]. The most important HF risk factor related to outcome in DM patients is a higher value of glycosylated hemoglobin (HbA_1c_), although a strict glycemic control is not associated with a substantial decrease in CV events [6]. These data underline the importance of cumulative glycemic burden, rather than glycemic control at a given point in time [7,8]. The high incidence of HF and its poor prognosis have been linked to the presence of an underlying diabetic cardiomyopathy (DCM), characterized by myocardial fibrosis and parietal stiffness. DCM is associated with depressed mechanical function, advanced glycation end products (AGEs) accumulation, defects in sub-cellular organelles and catecholamine receptor down-regulation. Due to the paucity of clinical trials, several questions are currently arising about the exact role of diabetes in HF development, its prevalence in HF patients, the different outcome in diabetic HF compared to non-diabetic patients, and the occurrence of dilated vs. concentric remodeling in DCM phenotypes. Finally, myocyte cells transduction signals and their metabolisms have been recently involved in the DCM development, although precise mechanisms remain to be investigated. Overall, whether diabetes could be considered a causal factor or simply a comorbidity in HF remains to be clarified. Despite these data, a specific trial evaluating the impact of diabetes in HF and the incidence of DCM in the diabetic population does not exist.

## 2. Epidemiology of Diabetes in Patients with Heart Failure

The prevalence of DM in HF patients ranges from 10 to 30%, reaching 40% in acute hospitalized patients, and these numbers are expected to grow exponentially over the next decades with the increase of an aging population [9]. DM is an independent predictor of CV morbidity and mortality in patients with chronic symptomatic HF, both in those with preserved ejection fraction (HFpEF) and in those with reduced ejection fraction (HFrEF), revealing a similar diagnostic impact [10]. However, the prevalence of diabetes in clinical trials ranges considerably in relation to diabetic type 2 or 1 and an acute or chronic HF setting: in some clinical trials of type 2 antidiabetic drugs, the prevalence of HF at baseline has varied between 10% and 30%. Interestingly, in chronic HF patients, the prevalence of DM was around 30% regardless of HFrEF and HFpEF. The highest prevalence of DM was registered in trials of acute HF (around 40%) [9]. Diabetes is also associated with an elevated risk of hospitalization ~33% [11]. Conversely, the presence of HF is an independent risk for developing type 2 diabetes. During a three-year follow-up of HF patients without diabetes, 29% versus 18% of matched controls developed diabetes and HF was shown to be an independent risk factor for diabetes occurrence [12]. Current risk seems to be further increased in the elderly population with HF: during a four-year follow-up there was a 4.78-fold (95% CI 1.8–12.4; *p* = 0.001) increased risk of developing type 2 diabetes.

There is wide heterogeneity in the prevalence of DM among different ethnic groups with HF. The prevalence of DM was lowest in whites (29.3%), followed by Japanese/Koreans (34.1%), blacks (35.9%), Chinese (42.3%), Indians (44.2%), and highest in Malays (51.9%) [13]. Therefore, the risk of adverse events ranges in relation to the antidiabetic treatment: rates of death from any cause and hospitalization are higher in all patients taking insulin compared to antidiabetic therapy in a dataset including 24,000 patients. Insulin prescription was associated with a higher risk of all-cause death [odds ratio (OR) 2.02, 95% confidence interval (CI) 1.87–2.19] and rehospitalization for HF (OR 1.42, 95% CI 1.32–1.53) [14]. DM is associated with an increased risk of morbidity and mortality in patients with chronic HFrEF, whereas clinical trial data suggest that the negative prognostic association with DM may be greater in HFpEF than in HFrEF. Data from the I-PRESERVE Trial showed that diabetic patients had an increased risk of CV death or HF hospitalization [Hazard Ratio (HR) 1.75, 95% CI 1.49–2.05; HR 1.59, 95% CI 1.33–1.914] [15]. Therefore, a recent analysis from large clinical trials of HFpEF demonstrated that patients with DM and HFpEF have greater morbidity and long-term mortality than those without DM. Besides, findings from the GWTG-HF registry show that in HFpEF, DM is associated with worse in-hospital and post-discharge morbidity, specifically longer length of stay, and increased 30-day all-cause and HF readmissions [16]. The impact of DM and pre-DM was also analyzed in 6935 patients with chronic HF in the GISSI Trial. Compared to non-DM subjects, those with DM had remarkably higher incidence rates of all-cause death (34.5% versus 24.6%). Conversely, both event rates were similar between non-DM patients and pre-DM patients [17]. Despite these data, in the post-hoc analysis of the EVEREST trial in patients with systolic dysfunction discharged with acute HF, DM was associated with the combined end point of cardiovascular mortality and HF hospitalization (HR 1.17; 95% CI 1.04–1.31) after adjusting for confounding factors [18]. Finally, in the PARADIGM study, the HbA_1c_ measurement showed an additional 13% of patients with undiagnosed DM and 25% with pre-DM. The HR for patients with undiagnosed diabetes mellitus (HbA_1c_, > 6.5%) and known diabetes mellitus compared with those with HbA_1c_ < 6.0% was 1.39 (1.17–1.64) and 1.64 (1.43–1.87) respectively [19]. In the last meta-analysis including 12 studies, patients with both acute and chronic HF, DM was associated with a higher risk of all-cause death (random-effects hazard ratio [HR] 1.28 [95% CI 1.21, 1.35]), cardiovascular death (1.34 [1.20, 1.49]) and hospitalization (1.35 [1.20, 1.50]). The impact of diabetes on mortality and hospitalization was greater in patients with chronic HF than in those with acute HF [20].

## 3. Impact of Diabetes on Heart Failure Occurrence

Despite the fact that it has been amply demonstrated that DM is associated with a three-fold increase in cardiovascular mortality due to micro and macrovascular complications, few data are reported about the impact of DM in HF occurrence. Notably, since hospitalization for HF is one of the most important complications in DM, trials on glucose lowering drugs should take into consideration this feature as a pre-specified primary end point. Therefore, the reduction of HbA_1c_ by common antidiabetic treatments, is acknowledged as a surrogate marker of CV disease reduction but epidemiological studies showed only a modest improvement in CV outcome. The third concern is about the role of DM as a causal factor or complication in HF: accordingly, many patients with diabetes have a higher prevalence of HF and at the same time, DM is also a potential cause for HF development [21]. Consequently, there is an impelling need to study DM taking all these concerns into account, particularly in large scale population studies that consider HF in patients with DM as a primary end point despite the presence of other cardiovascular diseases. The first population-based trial that investigated this aspect is the Framingham Trial revealing that DM subjects had a 5-fold increased risk of developing HF, and this trend was even worse when patients with known CAD were excluded [22]. A prospective observational study demonstrated that the DM ranged from 24–40% in HF patients and one third had HFpEF [23]. More recently, the Health ABC trials, during a long follow-up period of 11 years, showed that 16% of the enrolled population developed diabetes with an incident rate for HF occurrence of 2.5% per 100 person-years [24]. Current data imply that HF development is not necessarily due to the increased atherosclerotic burden and that DCM plays a relevant role in this field. Similar findings were revealed by Nichols et al, in which incidence of HF in patients with type 2 DM increased 3.3% per year, and 12% of included patients had some signs of HF at admission [25]. In the UKPDS program, people with DM showed an incidence for HF occurrence of 3 per 1000 person-years. Such incidence substantially increased when DM was combined with chronic renal dysfunction and anemia up to 44 per 1000 person-years [26,27]. Besides, in elderly patients, mortality in DM increased five-fold compared with subjects without DM [28]. High incidence of HF in DM patients strengthens the concept of ventricular dysfunction and structural myocyte alteration even in the absence of evident cardiac remodeling. Indeed, echocardiographic studies in apparently asymptomatic diabetic patients, found high percentages of diastolic dysfunction by tissue Doppler analysis, even in the absence of clear systolic dysfunction. The incidence of HF in these subjects appears strictly related to glycemic control and an improvement of HbA_1c_ results in 10% mortality reduction in HF related causes [29]. In the Olmstad County Minnesota registry, more than half of asymptomatic diabetic patients experienced diastolic dysfunction [30]. Another important issue is related to the glucose lowering treatment: a recent analysis of the European population, showed that insulin administration could impair prognosis compared to oral therapy [14]. Nevertheless, the ORIGIN trial including 12,000 patients did not demonstrate significant differences in terms of HF episodes between subjects submitted to insulin glargine and controls [31]. Taken together, all these notions raise several doubts regarding the real prevalence of HF in DM patients, which type of treatment could be more useful to prevent CV and HF complications, and the impact and incidence of DCM in either asymptomatic and decompensated patients. 

## 4. Pathophysiological Mechanisms of Diabetic Cardiomyopathy: from Molecular to Structural Dysfunction

Traditionally, the most important cause of mortality in diabetic patients has been attributed to CAD related events. However, a number of people with diabetes have HF even in the absence of coronary vessel atherosclerosis and in the presence of normal blood pressure values. Indeed, DCM often manifests a long subclinical period in most patients, before causing symptoms [32]. Notably, DCM proceeds in three different stages: the asymptomatic (early) stage, which is characterized by a normal systolic function with the onset of compensatory cardiac hypertrophy; the diastolic dysfunctional stage and the systolic dysfunctional (late) stage. Several molecular and cellular mechanisms are thought to explain DCM development. The heart is a continuously working engine that needs substrate products to maintain its contractile function. In resting condition, long-chain fatty acid beta-oxidation is the preferred energy substrate of the myocardium. 70% of energy expenditure comes from fatty acid oxidation whereas, glucose utilization, lactic acid, ketones and amino acids contribute to the remaining 30%. The uptake of free fatty acids (FFA) is mediated by the cluster of differentiation 36 (CD36), while glucose intake occurs through GLUT4. When insulin resistance occurs CD36 becomes preferentially localized to the sarcolemma, whereas GLUT4 is internalized and returns to its intracellular location [33]. Alterations in the fatty acid metabolism can contribute to cardiac dysfunction [34]. Many pathophysiological mechanisms are responsible for cardiac remodeling and dysfunction: hyperglycemia, lipotoxicity, microvascular AGEs deposition, autoimmunity, insulin resistance and hyperinsulinemia. In particular hyperglycemia, insulin resistance and hyperinsulinemia are considered independent risk factors for the development of DCM. The main vascular alterations associated with hyperglycemia include endothelial dysfunction, vascular effects of AGEs, adverse effects of circulating free fatty acids (FFA) and increased systemic inflammation. 

**Figure 1:**
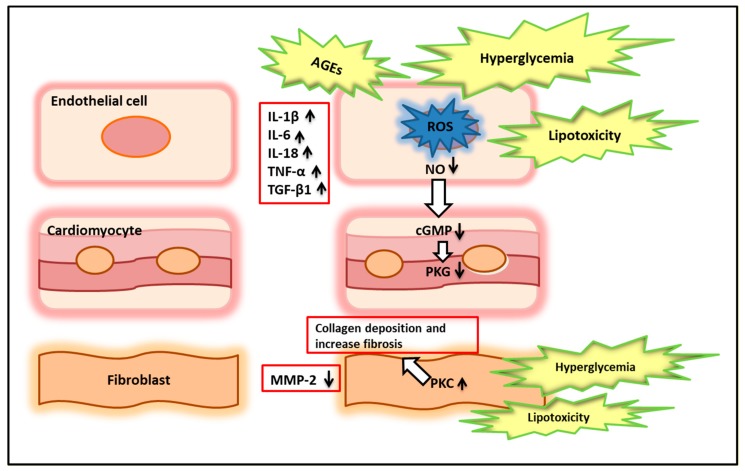
Cardiovascular alterations and signal dysfunctions in diabetic cardiomyopathy. AGEs: advanced glycation end products; ROS: reactive oxygen species; NO: nitric oxide; cGMP: cyclic guanosine monophosphate; PKG: protein kinase G; PKC: protein kinase C; MMP-2: matrix metalloproteinase 2; IL: interleukin; TNF-α tumor necrosis factor alfa; TGF-β1: transforming growth factor beta 1.

**Hyperglycemia**—When cells are exposed to high glucose concentrations, a loss of mitochondrial networks occurs with increased reactive oxygen species (ROS) and nitrogen (RNS) production. The mitochondrial electron transport chain is one of the first targets of high glucose levels, with a direct increase in superoxide anion formation. Increased levels of superoxide anion are driven by a vicious circle involving glucose-induced activation of protein kinase C (PKC) [35,36]. Activation of PKC by glucose leads to up-regulation of nicotinamide adenine dinucleotide phosphate (NADPH) oxidases, xanthine oxidase, uncoupling of NO synthase, microsomal P-450 enzymes and the arachidonic acid metabolism pathways. The production of superoxide anion reacts with nitric oxide (NO) released by endothelial nitric oxide synthase (eNOS) [37]. The reduced bioavailability of myocardial NO determines the inhibition of guanylyl cyclase and the reduction of cyclic guanosine monophosphate (cGMP) with a decrease in protein kinase G (PKG) activity. PKG activity reduction in cardiomyocytes causes hypophosphorylation of the giant cytoskeletal protein titin which controls diastolic myocardial distensibility [38]. Consequently, slow relaxation, high diastolic stiffness and reduction in cardiomyocyte elastance lead to diastolic dysfunction [39]. Moreover, hyperglycemia and lipotoxicity raise protein kinase C (PKC) activity in fibroblast, which results in interstitial deposition of collagen and reactive fibrosis [40]. All these cardiovascular alterations and signal dysfunctions are shown in Figure 1. 

**Hyperinsulinemia**—One of the main alterations in DCM is the impaired insulin signaling. In physiological conditions, there are two insulin signal transduction pathways. With the first one, insulin binds to the insulin receptor (IR) and activates insulin receptor substrates (IRS1 and 2) and subsequently downstream phosphoinositide-3-kinase (PI3K)-protein kinase B (Akt) pathways. Akt activation stimulates the translocation of GLUT4 to the cell membrane and the uptake of glucose into cardiomyocytes, skeletal muscle, liver, adipose tissue to maintain glucose homeostasis [33]. PI3K–Akt can also activate eNOS through direct phosphorylation at Ser^1177^, resulting in increased eNOS activity and NO production [41]. The resulting increase in bioavailable NO mediates coronary vasodilation and myocardial energy homeostasis. Cardiac insulin signaling dysregulation in diabetic patients determines impaired NO-mediated vascular relaxation, increased cardiac fibrosis and cardiac stiffness and causes impaired diastolic relaxation [42]. Excessive nutrient intake and inappropriate renin-angiotensin-aldosterone system (RAAS) activation determine insulin resistance through activation of the mTOR-S6K1 signal transduction pathway and the consequent attenuation of PI3K-Akt. The reduction of glucose uptake resulting from PI3K/Akt impairment, decreases Ca^2+^ ATPase activity and moves Ca^2+^ back into the sarcoplasmic reticulum, thus increasing intracellular Ca^2+^, with consequent contractile dysfunction and impaired relaxation. The second pathway involves signal transduction via mitogen-activated protein kinase (MAPK) [41], that regulates vascular function though stimulation of the expression of the vascular cell adhesion molecule (VCAM)-1 and the E-selectin on endothelium [43]. 

**FFA**—In DM, the energy contribution of glucose is greatly reduced in favor of beta-oxidation of FFA. In a state of insulin deficiency, adipose tissue lipolysis is enhanced, resulting in an elevated circulating FFA. Their increased utilization may have deleterious effects on myocardial function with abnormally high oxygen demand, intracellular accumulation of potentially toxic intermediates of FFA, and inhibition of glucose oxidation [44,45]. It should also be remembered that mitochondrial oxidative phosphorylation is a biochemical cellular process which provides 90% of intracellular ATP production in cardiomyocytes. In DM, mitochondria switch from glucose to FFA oxidation for ATP production. This leads to increased mitochondrial ROS generation and impaired oxidative phosphorylation. Altered mitochondrial Ca^2+^ handling further promotes mitochondrial respiratory dysfunction leading to cell death [46]. Metabolic stress-induced mitochondrial dysfunction also increases Ca^2+^ overload-induced opening of the mitochondrial permeability transition pores resulting in cardiomyocyte autophagy and cardiac necrosis [47].

**Advanced glycation end products**—AGEs are proteins or lipids that become glycated after exposure to sugars, which alters their functional properties [48]. The presence and accumulation of AGEs in many different cell types affect extracellular and intracellular structure and function. AGEs contribute to a variety of microvascular and macrovascular complications through the formation of cross-links in collagen molecules. This compromises the ability of collagen to be degraded, leading to increased fibrosis with subsequent increased myocardial stiffness and impaired cardiac relaxation [49]. AGEs activity causes an up-regulation of the receptor for advanced glycation end products (RAGE) and increasing oxidative stress. Activation of RAGE involves nuclear factor-κB (NFκB) and its target genes, which determines the shift from the alpha to the beta isoform of the myosin heavy chain in the cardiomyocytes of diabetic rats, responsible for reduction of myocardial contractility during HF [50]. Besides, AGEs block nitric oxide activity in the endothelium and cause the production of reactive oxygen species. 

**Inflammation**—Moreover, inflammation plays an important role in the pathophysiology of DCM [51,52,53]. Increased ROS in response to FFA and hyperglycemia activates NFκB, leading to an increased expression of cell adhesion molecules (ICAM-1) and vascular cell adhesion molecule 1 (VCAM-1), an increased infiltration of macrophages and leucocytes and an increased expression of inflammatory cytokines (IL-1β, IL-6, IL-18, TNF-α and TGF-β1) [54,55,56,57].

Increased oxidative stress is associated with the stimulation of various serine/threonine kinases c-Jun NH2-terminal kinase (JNK), PKCs, and IκB kinase complex β (IKK β) [58,59]. Their activation leads to serine phosphorylation of IRS-1, decreased activity of PI-3 kinase and Akt, which results in decreased GLUT4 translocation and glucose transport [43]. Thus, proinflammatory cytokines may contribute to insulin resistance by modulating insulin signaling and transcription. In addition, ROS activates transcription factors NFκB and activator protein (AP)-1 [60], which regulate transcription of proinflammatory genes, including interleukin (IL)-6, IL-1β, and tumor necrosis factor (TNF)- α. NFκB also stimulates the expression of adhesion molecules, including ICAM, VCAM, and E-selectin, which contribute to vascular pathology [61].

**Fibrosis**—Hyperglycemia-induced oxidative damage may play an important role in this pathogenetic process. Increased collagen deposition responsible for fibrosis may be related to an increased expression of transforming growth factor beta (TGF-β) and connective tissue growth factor (CTGF) or may be related to an increased activation of poly ADP-ribose polymerase 1 (PARP-1) in response to oxidative stress [62]. Furthermore, the reduced expression of some matrix metalloproteinases (MMPs) dysregulates extracellular matrix degradation with increased fibrosis [63].

**Impaired calcium handling**—The contraction mechanism is due to the interaction between actin–myosin mediated by calcium (Ca^2+^) homeostasis: Ca^2+^ influx via L-type Ca^2+^ channels triggers the release of Ca^2+^ from the sarcoplasmic reticulum. Conversely, relaxation occurs when Ca^2+^ is actively re-imported into the sarcoplasmic reticulum by SERCA-2a. In a mouse model of DM, the intracellular resting Ca^2+^ concentrations were elevated, intracellular Ca^2+^ decay was prolonged, SERCA-2a activity decreased and sarcoplasmic reticulum Ca^2+^ reuptake was damaged. All these features, translated into a human model, could hypothesize a specific role of Ca^2+^ overload in contractile dysfunction and in impaired relaxation in DCM [64,65]. 

**Figure 2:**
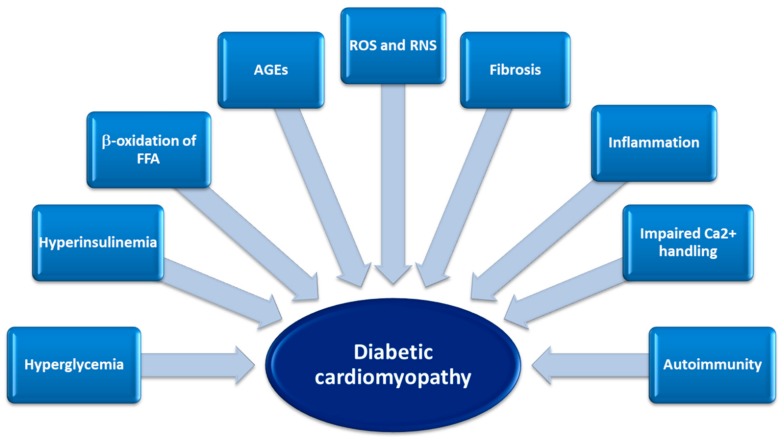
Contributing factors, metabolic derangement and molecular alterations in the development of diabetic cardiomyopathy. FFA: free fatty acids; AGEs: Advanced glycation end products; ROS: reactive oxygen species; RNS: reactive nitrogen species.

**Autoimmunity**—Finally, autoimmunity plays an important role in DCM. Gottumukkala et al. observed a severe post-infarction autoimmune syndrome in patients with type 1 DM, characterized by destructive lymphocytic infiltrates in the myocardium, cardiac autoantibody production and T helper type 1 effector cell responses against cardiac myosin. The authors have shown that patients with type 1 DM frequently develop persistent cardiac autoimmune responses following myocardial infarction. These autoantibodies may indicate ongoing “active” myocarditis that potentially upset myocardial fiber geometry and increased cell death. At the same time, the persistent release of troponin observed in these patients, could lead to an effective cardiac muscle mass reduction leading to the progression of systolic dysfunction in chronic DCM [66,67]. Both metabolic derangement and molecular alterations were involved in DCM development, as shown in Figure 2.

## 5. MicroRNA Signals in Diabetic Cardiomyopathy

In recent years, microRNAs (miRs), a class of non-coding-RNA, have been proposed to facilitate the diagnostic process. MiRs have been described as the “micromanagers” of gene expression because they exercise post-transcriptional control over most genomes and several of them have shown a strong relation to cardiomyocyte cell death of the diabetic heart. MiRs are subjected to various processes before maturation. MiRs genes are usually transcribed by RNA polymerase II to form primary-microRNA (pri-miR). This is cleaved further by an endonuclease enzyme, Drosha, together with its essential cofactor DiGeorge Syndrome Critical Region gene 8 (DGCR8), to form long precursor-microRNAs (pre-miRs). Pre-miRs are next exported to the cytoplasm with the help of a transporter protein, Exportin 5, which is further cleaved by endonuclease enzyme, Dicer, into small interfering RNA (siRNA) like an imperfect duplex comprising one mature-miR strand, which is incorporated into ribonucleoprotein and a complementary sequence which is degraded. Many authors analyze miRs expression in the diabetic heart and also in HF it is classified on the basis of ejection fraction in HFpEF and HFrEF [68,69,70]. Costantino et al. showed that 316 miRs were dysregulated in the DCM; among them, miR-221 and miR-212 were massively over expressed in these patients and were related to hypertrophy and autophagic response. Similarly, miR-34a, miR-195, miR-1/206, miR-320, miR-378 and miR451 were related to cardiomyocytes apoptosis when upregulated [71,72,73,74]. Moreover, other micro-RNAs such as miR-1, miR-373, miR-378 and miR-133a were downregulated in case of hypertrophy development and oxidative stress increase [69,75]. These findings confirm the potential role of these modulators in identifying DCM, in discovering phenotype occurrence, and possibly, to target a treatment on the basis of miRs findings and changes over time. From the preliminary data, it is conceivable that down or up-regulation of certain miRs are involved in molecular mechanisms and that signal transduction dysfunction is involved in DCM development. The different phenotypes should express different causative processes such as systemic inflammation, myocytes hypertrophy and fibrosis rather than cardiomyocytes apoptosis and death mediated by immune reaction. Moreover, the persistent alteration of miRs induced by high blood glucose values could explain the progression of DCM despite the normalization of glycemic levels, suggesting the presence of hyperglycemic memory in the diabetic heart. The integration and crossover of biomolecular and imaging data could be of interest for early recognition of DCM and its complex pathophysiological network. Therefore, the over and under expression of laboratory findings could improve the acknowledgement of vascular myocyte and endothelial injury, the relative signal**s** transduction alterations and their comparison in concentric and dilatative cardiomyopathy pattern**s**. Finally, miRs are able to remain stable in the circulation during transfer between the cells or to the tissues, making them an up-and-coming diagnostic, prognostic and therapeutic marker. Differential influence of miRs on DCM phenotypes would be deeper investigated. Table 1 shows the main miRs upregulated or downregulated in DCM. 

## 6. Different Phenotypes in Diabetic Cardiomyopathy

**Figure 3:**
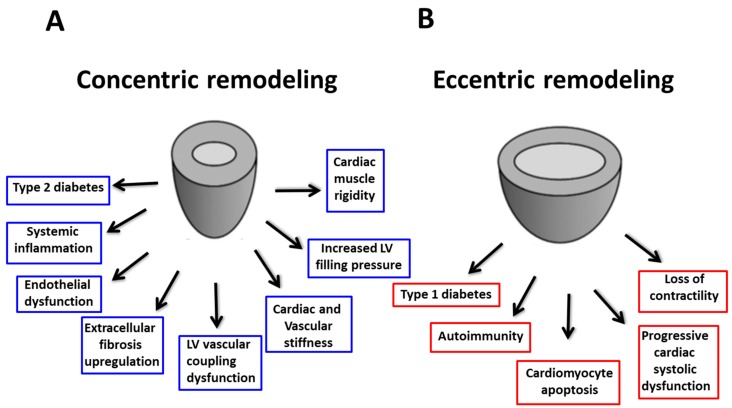
Different phenotypes in diabetic cardiomyopathy: (**A**) concentric remodeling due to increased fibrosis, cardiovascular stiffness and LV chamber rigidity. (**B**) eccentric remodeling due to cell apoptosis, progressive cardiac enlargement and systolic impairment. LV: left ventricular.

Although the pathogenesis of DCM remains controversial, several molecular and cellular alterations have been hypothesized: myocardial hypertrophy, interstitial fibrosis, oxidative stress and vascular inflammation are all potential mechanisms leading to increased LV enlargement, increased stiffness, reduced compliance with consequent diastolic dysfunction and cardiac contractility impairment. In 1954, Lundbaek was the first to describe myocardial dysfunction as a complication of diabetes [76]. Two decades later, Rubler et al. described a myocardial disease characterized by a dilated phenotype with eccentric LV remodeling and systolic dysfunction secondary to diabetic microangiopathy in the absence of major CAD [77]. In 2011, Maisch et al. proposed a classification of DCM. They divided DCM into four stages: stage 1 included diastolic HFpEF, with hypertrophic and restrictive phenotype; stage 2 included diastolic and systolic HFrEF with dilatation phenotype. In these two classes, CAD and hypertension did not play a role. Instead, stage 3 included DCM with diastolic and systolic dysfunction in which hypertension, microangiopathy and viral heart disease played a role and stage 4 included all confounding factors, such as CAD [78]. More recently, a new paradigm regarding DCM has been accepted [1,10,79,80]. In 2013, the American College of Cardiology Foundation (ACCF), the American Heart Association (AHA) [81] and the European Society of Cardiology (ESC) in collaboration with the European Association for the Study of Diabetes (EASD) [82] defined DCM as a clinical condition of ventricular dysfunction that occurs in the absence of coronary atherosclerosis and hypertension in patients with diabetes. DCM could recognize two different phenotypes according to different molecular adaptation and damage [1]. The first phenotype of DCM described is characterized by concentric hypertrophy without cardiac chamber dilatation, LV diastolic dysfunction with preserved systolic function, increased myocardial stiffness and elevated LV end-diastolic pressure [83]. More specifically, DCM is related to increased systemic inflammation mediated by hyperglycemic status, insulin resistance, lipotoxicity and interstitial deposition of AGEs. The systemic inflammation triggers myocardial microvascular endothelial activation with the expression of adhesion molecules (ICAM, E-selectin). These mechanisms lead to the structural modification of microvascular circulation, such as thickening capillary basement membrane, microvessel obstruction and microvascular rarefaction. The activation of microvascular coronary arteries favors myocardial infiltration by activated macrophages modulated by oxidate LDL. Simultaneously, the activation of inflammatory cytokines alters paracrine signaling between endothelial cells and cardiomyocytes with subsequent myocardial infiltration by inflammatory cells. Leukocytes infiltration promotes myofibroblasts activation and interstitial collagen deposition. Hypertrophy and interstitial fibrosis represent the final product of the inflammatory cascade with HFpEF development. According to these findings, several authors have recently demonstrated the relation to several inflammatory/fibrosis biomarkers such as IL-1β, TNF-α, IL-18, IL-6, TGF-β and Galectin-3 in the concentric DCM isoform. In this setting a recently proposed biomarker, called ST-2, demonstrated a good prognostic power. Soluble ST-2 has been identified as the ligand for interleukin-33 (IL-33) and it exists in two isoforms: a transmembrane and a soluble variety. IL-33/ST-2 ligand complex has anti-proliferative and anti-fibrotic properties. On account of its expression in cardiac myocytes, endothelial cells and fibroblasts, the soluble form has been identified as a promising biomarker in acute myocardial infarction and remodeling predictions as well as in decompensated HF [84]. The second proposed DCM phenotype was the dilated pattern, which is characterized by LV eccentric remodeling with reduced systolic function. HFrEF DCM results from cardiomyocyte cell death usually caused by ischemic heart disease. In particular, in HFrEF DCM, the up-regulation of the free-radical producing enzyme nicotinamide adenine dinucleotide phosphate oxidase (NOX2) was observed in cardiomyocytes secondary to ischemic injury. The enhancement of cardiomyocytes FFA uptake in DM leads to mitochondrial dysfunction, which is another important cause of cardiomyocyte death. This process proceeds together with the increase of proapoptotic genes expression, which is linked to cardiomyocytes death. Two other important mechanisms associated with HFrEF DCM are fibrosis replacement and autoimmunity. Thus, lipotoxicity or activation of NFkB by AGEs together with hyperglycemia are the main actors of fibrosis replacement, overall through PKC activity in fibroblast. In this sense, several biomarkers are higher in this phenotype. NTproBNP and high sensitivity troponin T, respectively biomarkers of myocardial wall stress and cardiomyocyte injury, showed a higher accuracy in recognizing HFrEF patients with eccentric LV remodeling [1,10,79,80]. Figure 3 shows the different mechanisms leading to the two phenotypes. 

Finally, the last studies no longer consider DCM as a dichotomous phenotype, but rather as a progression from an early asymptomatic stage to the diastolic dysfunctional stage and therefore to the systolic one.

Although several mechanisms have been recognized in the pathogenesis of DCM, there are few data available about the main pathophysiological process involved in DCM regarding the concentric versus dilated patterns. Furthermore, the phenotypes and the underlying mechanisms of DCM have been mostly researched in db/db mice, ob/ob mice, Zucker diabetic fatty rats, and less studied in diabetes patients. These results should be translated into human population in order to confirm preliminary animal data. Accordingly, further investigations and future researches may be warrant.

## 7. Conclusions

DCM is an emerging disease, responsible for HF episodes in at least 50% of diabetic patients. This syndrome has been overlooked too often in the past and it deserves specific diagnostic and clinical care. It could appear in two main patterns depending on the underlying prevalent pathophysiological mechanism, myocyte alteration, and molecular substrate. Because of the lack of specific studies, several questions remain to be elucidated: the exact role of DCM in HF occurrence, its prevalence in the whole diabetic population, and the appearance of dilated vs. a concentric remodeling phenotype. Future studies are needed to clarify all these aspects and to identify the structural and molecular alterations of this new entity in the future. 

## Figures and Tables

**Table 1:** Role of main miR candidates in DCM mechanisms and pathophysiology.

miRs	Expression Pattern	Pathophysiological Role	Expression Sight
miR-1 [69,75]	Downregulated	Hypertrophy and oxidative stress	Cardiac and skeletal muscle
miR-1/206 [71]	Upregulated	Cardiomyocytes apoptosis	Cardiac muscle
miR-34a [71,74]	Upregulated	Cardiomyocytes apoptosis	Cardiac muscle
miR-133a [69]	Downregulated	Hypertrophy and oxidative stress	Cardiac and skeletal muscle
miR-195 [71]	Upregulated	Cardiomyocytes apoptosis	Cardiac muscle
miR-212 [71,73]	Upregulated	Hypertrophy and autophagic response	Cardiac and skeletal muscle
miR-221 [71,72]	Upregulated	Hypertrophy and autophagic response	Cardiac and skeletal muscle
miR-320 [71]	Upregulated	Cardiomyocytes apoptosis	Cardiac muscle
miR-373 [69]	Downregulated	Hypertrophy and oxidative stress	Cardiac muscle, endothelium
miR-378 [71]	Downregulated	Hypertrophy and oxidative stress	Cardiac muscle, endothelium
	Upregulated	Cardiomyocytes apoptosis	Cardiac muscle
miR-451 [69]	Upregulated	Cardiomyocytes hypertrophy	Cardiac muscle
